# Construction of inflammatory associated risk gene prognostic model of NSCLC and its correlation with chemotherapy sensitivity

**DOI:** 10.1080/07853890.2023.2200034

**Published:** 2023-04-21

**Authors:** Yange Gong, Hongyan Pang, Zhiqiang Yu, Xue Wang, Ping Li, Qianyun Zhang

**Affiliations:** Department of pulmonary and Critical Care Medicine (PCCM) Ward II, Cangzhou Central Hospital, Cangzhou City, Hebei Province, China

**Keywords:** NSCLC, inflammation, chemotherapy, risk-score, KRT6A

## Abstract

**Background:**

Inflammation is an important pathogenic factor of most malignant tumors. It is essential to understand mechanism underlying inflammation and cancer development, so as to formulate and develop anti-cancer treatment strategies. However, inflammatory-related gene characterization as well as risk model construction in prognosis and response chemotherapy or immunotherapy in NSCLC are still remain unclear.

**Methods:**

A total of 1014 lung cancer samples with RNA-seqencing results were download from The Cancer Genome Atlas (TCGA) database. The patient cohort was randomized as a training and test cohorts, and 200 inflammatory-related genes were selected based on previously published data. Consensus clustering and Enrichment and immune function analyses base on Differential expression genes (DEGs) were performed. Prognosis Prediction Model were Constructed and Chemotherapy and immunotherapy sensitivity base on this model were performed. At last, H1299 and HCC827 cells were used to tested the mitoxantrone and oxal iplatin sensitivity after KRT6A knockdown.

**Results:**

We identified the inflammatory-related genes from NSCLC datasets to build one prognosis prediction signature based on cluster inflammatory-related genes to lay a certain foundation for distinguishing high-risk NSCLC cases with dismal prognostic outcome. The nomogram provides the AUC values for 1-, 3-, and 5-year overall survival were 0.831, 0.853, and 0.86 in validation cohort. Morover, different sensitivity of immunotherapy or chemotherapy also were classified base on the different risk groups in NSCLC patients, which provided potent clinical reference. At last, targeting KRT6A sensitive to mitoxantrone and oxaliplatin in H1299 and HCC827 cells.

**Conclusions:**

Inflammatory-related gene risk-score is the potential chemotherapeutic and immunotherapeutic biomarker for NSCLC, and targeting KRT6A sensitive to mitoxantrone and oxaliplatin in NSCLC.

1. Highlights
  Inflammatory-related genes can lay a certain foundation for distinguishing high-risk NSCLC cases with dismal prognostic outcome.
2. Risk-score base on inflammatory-related genes is positive correlated with CD274, TGFBR1 and TGFB1 expression.
3. Targeting KRT6A sensitive to mitoxantrone and oxaliplatin in H1299 and HCC827 cells.

## Introduction

Lung cancer, a heterogeneous disorder, can be divided as small cell lung cancer (SCLC) and non-small cell lung cancer (NSCLC). NSCLC represents the highly malignant neuroendocrine tumor that represents approximately 85%–90% of all lung cancers. It has the features of rapid metastasis, short tumor doubling time, together with poor 5-year survival [1,2]. However, latest advances in immunotherapy and targeted therapy have provided new avenues for precise cancer treatment in patients with NSCLC. Notably, immune checkpoint inhibitors (ICIs) can significantly improve NSCLC patient survival at the locally-advanced or metastatic stage [3]. Despite this progress, primary and acquired ICIs resistance are commonly seen among these cases [4]. Therefore, the identification of potential biomarkers for predicting responses to chemotherapy, targeted therapy and immunotherapy for NSCLC holds immense clinical significance.

As reported in many recent articles, inflammation is a critical factor leading to tumor occurrence and development [5]. Inflammation is the body’s immune defense mechanism against harmful irritants. It can reduce infection, remove damaged cells and start tissue repair [6]. Persistent inflammatory stimulation and immune cell activation can lead to long-term repeated tissue destruction and regeneration, resulting in chronic inflammation [7]. As molecular biology progresses and transgenic mouse technology develops, inflammation mechanisms at molecular and cellular levels during tumor occurrence, metastasis and progression are slowly revealed, including the functions of various immune cells and complex signal pathways coordinated by various cytokines, chemokines and growth factors [8–10]. In addition, the rise of new technologies represented by high-throughput sequencing (NGS) and single cell sequencing technology has further improved the theoretical framework in the field of cancer and inflammation [11,12]. Inflammation is an important pathogenic factor of most malignant tumors. It is essential to understand mechanism underlying inflammation and cancer development, which facilitates the formulation and development of anti-cancer treatment strategies. However, inflammatory-related gene characterization as well as risk models in prognosis and response chemotherapy or immunotherapy in NSCLC remain unknown.

The present work discovered inflammatory-related genes in NSCLC datasets to build a prognosis prediction model for the sake of laying a certain foundation for distinguishing high-risk NSCLC cases with dismal prognostic outcome. Morover, the different sensitivity of immunotherapy or chemotherapy also were classified base on the different risk groups in NSCLC patients, which provided potent clinical reference.

## Materials and methods

### Data extraction and processing

The Cancer Genome Atlas (TCGA) database (https://portal.gdc.cancer.gov/) was employed to acquire clinical and gene data of NSCLC cases. This dataset consisted of 513 tumor samples of lung adenocarcinoma (LUAD) and 501 tumor samples of lung squamous cell carcinoma (LUSC), as well as 108 normal samples. The patient cohort was randomized as one training cohort comprising 710 cases together with one test cohort comprising 304 cases. In order to explore potential markers of inflammation, 200 inflammatory-related genes were selected based on previously published data [13], which are presented in Supplementary Table 1.

### Consensus clustering and DEGs analysis

In this study, consensus analysis was carried out using the ‘ConsensusClusterPlus’ R package on TCGA-NSCLC cases using inflammatory-related genes, which facilitated the classification of patients into discrete clusters. This technique provides a quantitative measure for determining the best cluster number for RNA-seq data. Relation of identified clusters with clinical factors was evaluated and outcomes were displayed using a heatmap. Furthermore, we plotted Kaplan-Meier (K-M) curves for comparing prognoses of both identified clusters.

### Enrichment and immune function analyses

Differential expression genes (DEGs) were determined between distinct clusters of NSCLC patients using limma package, which were later filtered upon statistical significance thresholds of *p* < .05 and |log fold change (FC)| ≥ 1. Functional annotation of these DEGs was conducted using clusterProfiler, which encompassed Gene Ontology (GO) together with Kyoto Encyclopedia of Genes and Genomes (KEGG) analysis. Enrichment results were presented in a bubble diagram. Moreover, CIBERSORT algorithm was adopted for calculating immune scores, while cell infiltration levels were evaluated in NSCLC patients using the IOBR packages. The differences in immune infiltration between distinct clusters were examined.

### Prognosis prediction model construction

Univariate Cox regression was conducted by survival package for selecting prognostic genes for NSCLC. And intersection of DEGs and prognostic gene was taken. The differential prognostic genes with independent prognostic risk were further screened by multivariate analysis. They were then incorporated into the random forest prognostic model by randomForestSRC package. We used training cohort for model construction and validate against testing group. Time-dependent receiver operating characteristic (t-ROC) curves were plotted for evaluating our nomogram’s prediction performance with timeROC package. According to the bestcutoff of the randomForest (RF) scores of training cohorts, all cases were classified as low- or high-risk group.

### Construction of nomogram graph based on prognosis prediction model

As for if RF score was the prognostic predictor for OS independently, after comparing its clinical features, univariate as well as multivariate Cox regression was conducted. And ‘rms’ R-package builds a OS-based nomogram with prognostic features. AUC value is used to test how nomogram distinguishes survival. Constructing a nomogram with calibration curve to test survival at 1, 2 and 3 years based on nomogram and actual observations.

### Chemotherapy and immunotherapy sensitivity

For investigating different biological processes (BPs) of both groups, GSVA was carried out with ‘GSVA’ R package. We also calculated different RF scores of chemotherapy responders compared with non-responders in patients with TCGA NSCLC.

The OncoPredict R package for predicting sensitivity to drugs in tumor patients. In our study, We will compare different risk score groups to evaluate semi-maximum inhibitory concentration (IC50) values of drugs from tumor drug sensitivity genomics (GDSC; https://www.cancerrxgene.org/).

TIDE algorithm was utilized to assess tumor immune evasion opportunities from different risk-score [14]. We uploaded gene expression data from NSCLC tissues to TIDE’s online database (http://tide.dfci.harvard.edu/) for calculating different immune factors such as the TIDE value, dysfunction, and exclusion, which were adopted for predicting immune checkpoint blockade (ICB) therapeutic responses.

### Cell culture and lentiviral transduction

This work obtained H1299 and HCC827 cells from ATCC (Manassas, VA, USA) and cultivated them within RPMI-1640 medium that contained 10% FBS along with 1% penicillin-streptomycin (PS). To transfect and produce lentivirus, packaging plasmids were utilized to transfect to 293 T cells. The shKRT6A target sequence used was 5′-GCTCTCAAACTCTCTAACTTA-3′. Following this, stable KRT6A knockdown H1299 and HCC827 cells were selected *via* screening using puromycin.

## Immunofluorescence

To determine the expression of KRT6A, cells are formaldehyde fixation, dewaxed, hydrated, endogenous enzyme removal, and antigen retrieval. Subsequently, Anti-GPC3 antibody(1:50; Invitrogen, 10590-1-AP) and related fluorescent secondary antibody (ThermoFisher Scientific).

### Statistical analysis

R software (Version 4.0.3) was adopted for statistical analysis. Gene expression levels were compared by Wilcoxon rank-sum test between different clusters, and KM analysis and log-rank test were conducted for comparing overall survival (OS) of diverse groups. *p* < .05 (two-sided) stood for statistical significance unless otherwise indicated.

## Results

### Identification of two molecular subtypes according to inflammatory-related genes in NSCLC

**Figure 1.**
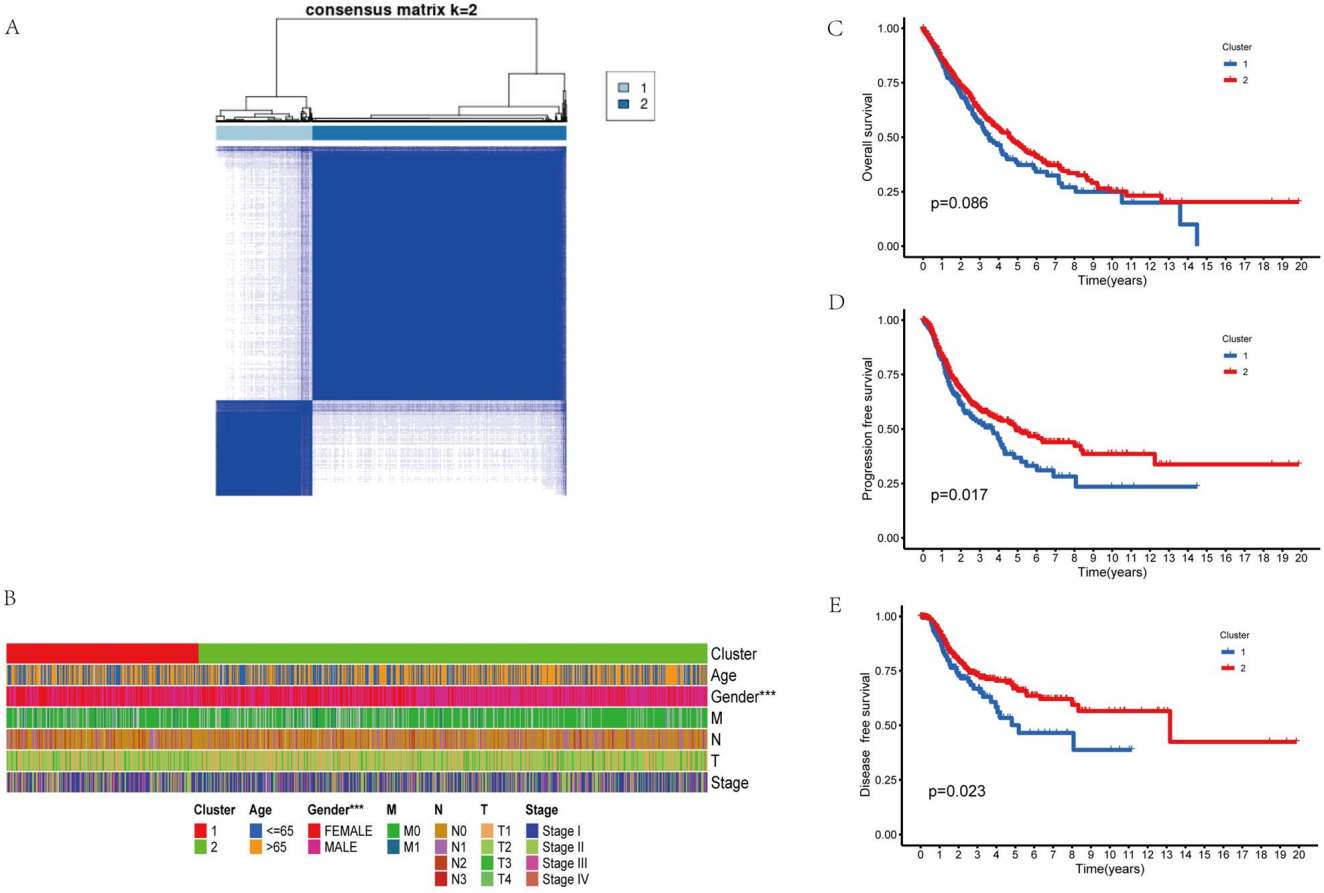
Identifification of two molecular subtypes base on inflammatory-related genes in NSCLC. (A) Molecular subtype discrimination of NSCLC patients base on inflammatory-related genes. (B) Clinical features comparison between the two clusters. (C–E) Prognostic value comparison between the two clusters in the NSCLS patients, include Overall survival, progression free survival and disease free survival.

NSCLC data were obtained based on TCGA, while patients were clustered using ConsensusClusterPlus package according to inflammatory-related genes. Patients were classified as two clusters (A, B) (Figure 1(A)). Clinical features of the two clusters included age, gender, TNM stage were shown in the Figure 1(B), differences in age and TNM stage were not significant in cluster A compared with cluster B. However, the cluster B has more ratio of male patients (Figure 1(C)). Interesting, OS, Disease-free survival (DFS) and progression-free survival (PFS) were not significantly different between the two groups (Figure 1(D)). Collectively, inflammatory-related genes might be adopted for distinguishing the NSCLC patient to two molecular subtypes with different prognosis.

### Enrichment analysis base on the inflammatory-related subtypes of NSCLC

**Figure 2.**
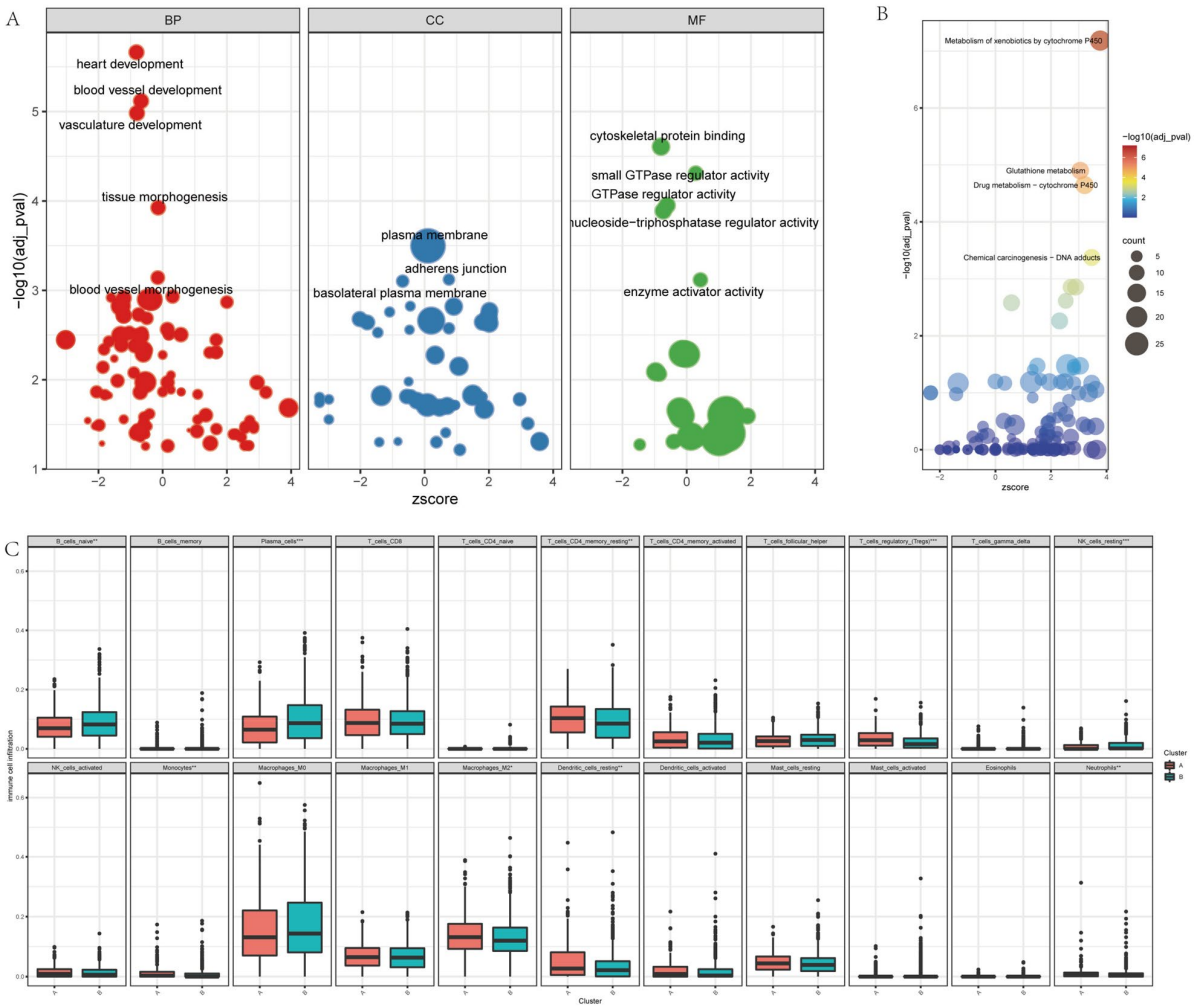
Enrichment analysis of the differences between the two clusters. (A) the GO (Gene Ontology) analysis base on the different genes of the two clusters, included biological process (BP), cell component (CP), and molecular function (MF). (B) KEGG (Kyoto Encyclopedia of Genes and Genomes) analysis base on the different genes of the two clusters. (C) Immune infiltration analysis with the CIBERSORT of the two clusters.

To further reveal the molecular characteristics of the inflammatory-related subtypes of NSCLC, we firstly enriched the BPs, molecular functions (MFs) and cell components (CCs) base on DEGs of both clusters *via* GO (Gene Ontology) analysis (Figure 2(A)). Interesting, we found vasculature development and vessel development are the main biological process between the clusters. The most different gene are belong to plasma membrane, adherens junction and basolateral plasma membrane related gene. For the MF analysis, the enriched genes are involve in cytoskeletal protein binding, GTPase regulator activity and small GTPase regulator activity. For KEGG analysis, the different genes are mainly take part in xenobiotics metabolism *via* cytochrome P450, drug metabolism-cytochrome P450, and glutathione metabolism (Figure 2(B)), these result further imply the inflammatory-related subtypes of NSCLC main correlated with the drug metabolism and resistance. In addition, we also compared the immune cell infiltrating degrees of both clusters. We found immune infiltration levels included naive B cells, resting Memory CD4+ T Cells, plasma cells, regulatory T cells (Tregs), resting NK cells, monocytes, M2 macrophages, neutrophils and resting dendritic cells are differents between both cohorts (Figure 2(C)). Therefore, these result shown different inflammatory-related subtypes of NSCLC with different biological process, molecular function, signaling pathway, as well as immune infiltration levels.

### Identify the critical genes base inflammatory-related subtypes of NSCLC patients

**Figure 3.**
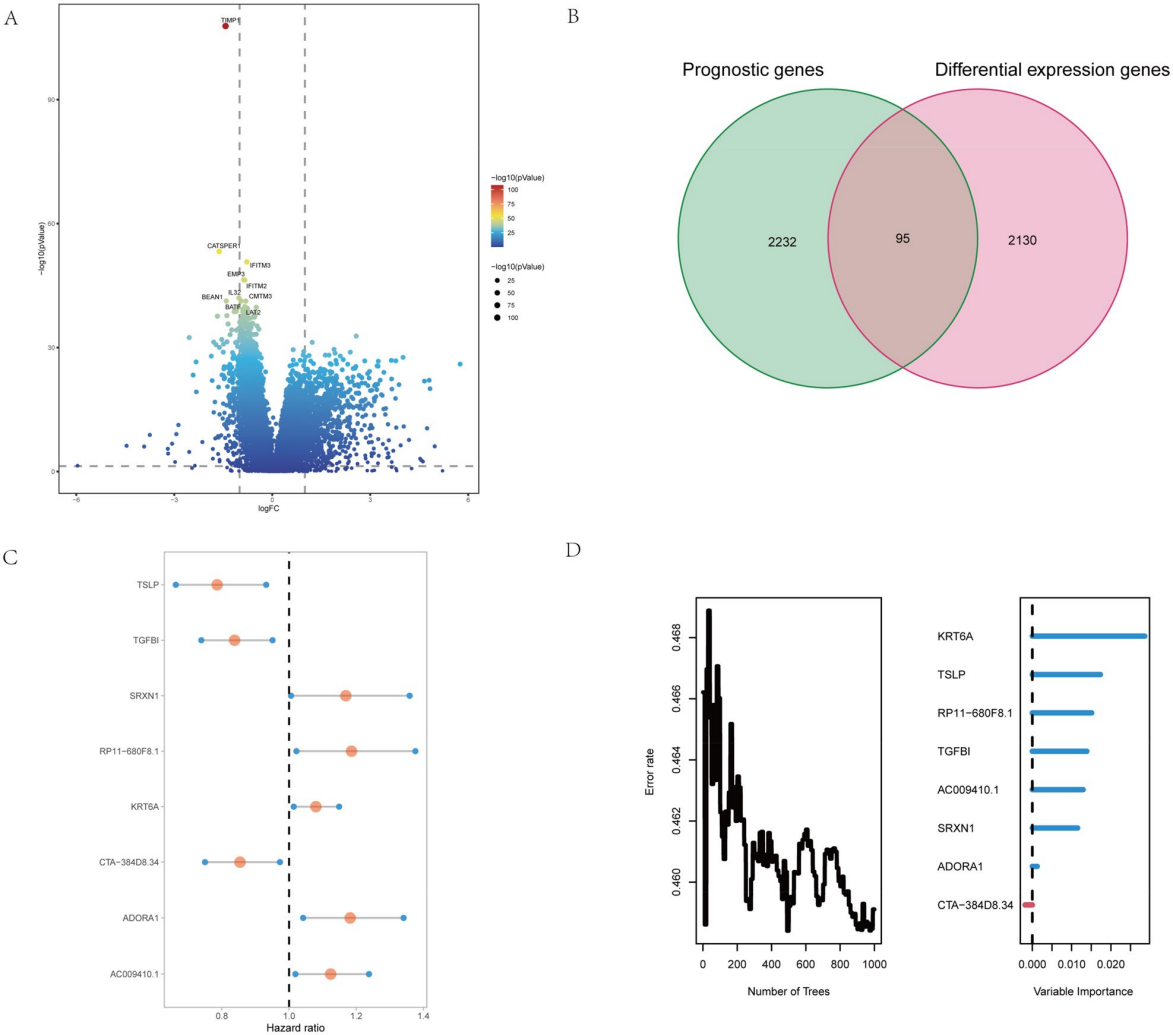
Identify difference expression and prognostic genes analysis between the two groups. (A) volcano map for Identification of difference expression genes of the two clusters. (B) the Venn diagram of difference expressed genes and prognostic genes for screen risk genes base on inflammatory-related genes. (C,D) By multivariate analysis of 95 differential genes, 8 independent differential genes were selected and included in the random forest prognosis model.

To further Identify the critical genes base inflammatory-related subtypes of NSCLC patients, we firstly compared the difference expression genes of both groups (Figure 3(A)). Moreover, we conduct the intersection genes with the difference expressed genes and prognostic genes for screening risk genes base on inflammatory-related subtypes of NSCLC patients (Figure 3(B)). We found 95 common genes, and further performed the multivariate analysis of 95 differential genes, 8 independent differential genes were selected and included in the random forest prognosis model (Figure 3(C,D)), included TSLP, TGFBI, SRXN1, RP11 − 680F8.1, KRT6A, CTA-384D8.34, ADORA1 and AC009410.1. Herein, we firstly identify the critical genes base inflammatory-related subtypes of NSCLC patients.

### Development of risk-score according to inflammatory-related gene model for NSCLC patients

**Figure 4.**
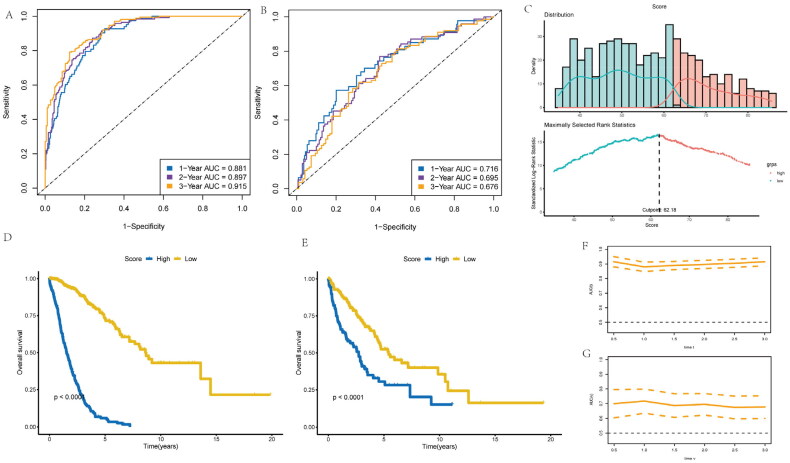
Development of risk-score based on the 8 inflammatory-related gene signature of NSCLC patients with the training cohort and the validation cohort. (A) ROC curve for 1, 3 and 5 years overall survival of the training cohort in NSCLC patients. (B) ROC curve for 1, 3 and 5 years overall survival of the validation cohort in NSCLC patients. (D) Kaplan–Meier survival plot showing overall survival using risk score of inflammatory-related gene signature in NSCLC training cohort. (E) Kaplan–Meier survival plot showing overall survival using risk score of inflammatory-related gene signature in NSCLC validation cohort. (F,G) Continuous AUC curve of 3-year in the training and validation cohort.

This work constructed one risk-score model to forecast NSCLC prognostic outcome using eight independent prognostic and inflammatory-related genes. This model employed the machine learning algorithm based on random forest (RF) method to analyze survival. Subsequently, the NSCLC patient’s survival was predicted, and area under the ROC curve (AUC) values were found to be 0.881, 0.897, and 0.915, respectively for 1, 3, and 5 years overall survival in the training cohort (Figure 4(A)). Similarly, those for validation set were 0.716, 0.695, and 0.616, separately (Figure 4(B)). Furthermore, cases were stratified as low- or high-risk group according to risk scores (Figure 4(C)), as a result, high-risk score cases exhibited poorer overall survival compared with low-risk score counterparts for training (Figure 4(D)) and the validation cohorts of NSCLC patients (Figure 4(E)). The AUC values based on different prognosis times of the two cohorts can be observed from Figure 4(F,G). Statistical analysis was performed with R software (version 4.0.3), with Wilcoxon test utilized to compare gene expression between different clusters. KM survival and log-rank test were utilized for comparing OS of both cohorts. *p* < .05 (two-sided) stood for statistical significance if not specified above.

### Construction and calibration of nomogram combined with clinical factors

**Figure 5.**
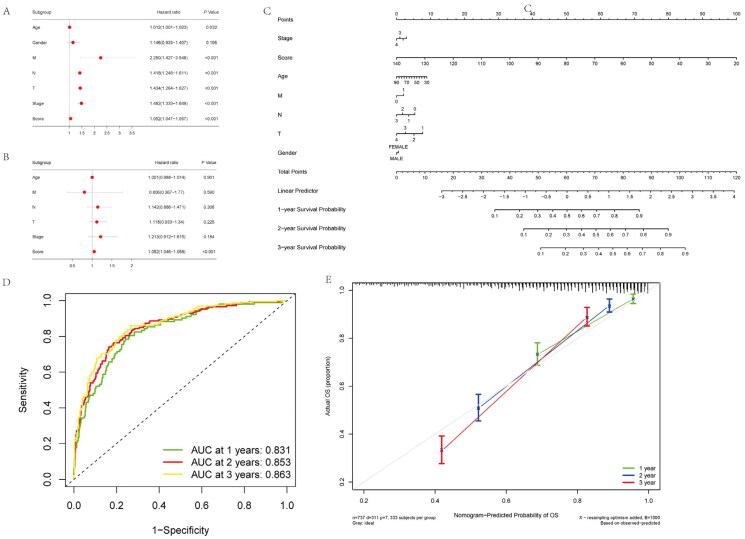
Construction and calibration of nomogram for prognostic prediction in NSCLC patients base on inflammatory-related gene signature. (A,B) Univariate and multivariate analysis on the risk score in NSCLC patients. (C) Nomogram based on the inflammatory-related gene signature NSCLC patients. (D) ROC curve of the Nomogram for 1, 3 and 5 years overall survival in the validation cohort in NSCLC patients. (E) Calibration curve of the nomogram at 1 year, 3 years and 5 years.

We conducted a multivariate analysis to assess whether a risk-score constructed by incorporating clinical characteristics was of predictive significance. As a result, risk score independently predicted prognosis of overall survival (OS) for NSCLC cases, based on age, gender, and TNM stage for two cohorts (Figure 5(A,B)). Using the training cohort, we trained a nomogram model in which the numeric scale displayed the score for each predicting factor, such as age, sex, risk score and stage. The sum of points for every predicting factor estimated the 1-, 3- and 5-year OS probabilities, as represented by the nomogram plot (Figure 5(C)).

In validation cohort, AUC values for 1-, 3-, and 5-year overall survival were 0.831, 0.853, and 0.863, separately, indicating good discriminative ability (Figure 5(D)). We also constructed calibration curves for assessing nomogram’s model performance. C-index in the prediction of OS of validation dataset was 0.798, 0.773, and 0.825 separately for years 1, 3, and 5, indicating that the nomogram-predicted value was well consistent with real measurement (Figure 5(E)). Therefore, our nomogram constructed by incorporating inflammatory-related gene risk score showed excellent prognostic value for NSCLC patients.

### Function, chemotherapy as well as immune infiltration analysis

**Figure 6.**
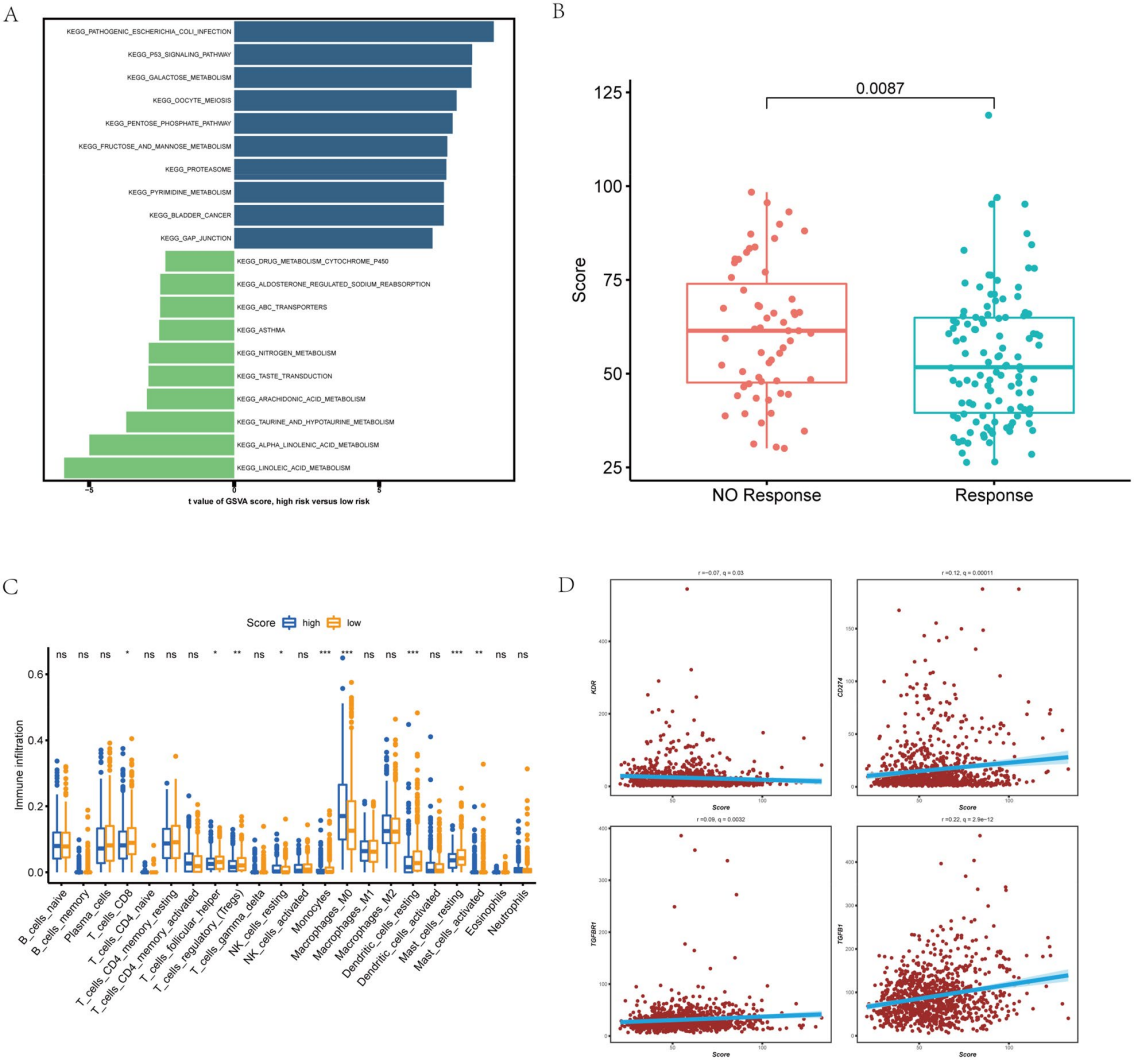
Functional, chemotherapy and immune infiltration analysis between different inflammatory-related gene signature. (A) Gene set variation analysis (GSVA) analysis is performed on patients with high and low scores. (B) The difference between response and non-response scores of NSCLC patients with chemotherapy in TCGA data. (C) Cibersort immune function analysis between the two groups. (D) correlation between immune marker and score.

For better revealing whether risk-score was of clinical value in NSCLC patients, we conducted gene set variation analysis (GSVA) for patients with high or low risk scores. GSVA result between two groups included P53 signaling pathway, drug metabolism-cytochrome P450 and galactose metabolism, as well as other pathways (Figure 6(A)). Moreover, high risk-score group has lower response scores of chemotherapy in NSCLC patients (Figure 6(B)). And patients with high risk-scores had lower CD8+ T cell and macrophages infiltration (Figure 6(C)). To evaluate the risk-score and immune escape in NSCLC patients, we found that risk-score is positive correlated with CD274, TGFBR1 and TGFB1 expression (Figure 6(D)). These data further reveal the NSCLC patients with different inflammatory-related gene risk-score may has different molecular pathway, immune infiltration level and response of chemotherapy.

### Inflammatory-related gene signature correlated with chemotherapy and immunotherapy resistance

**Figure 7.**
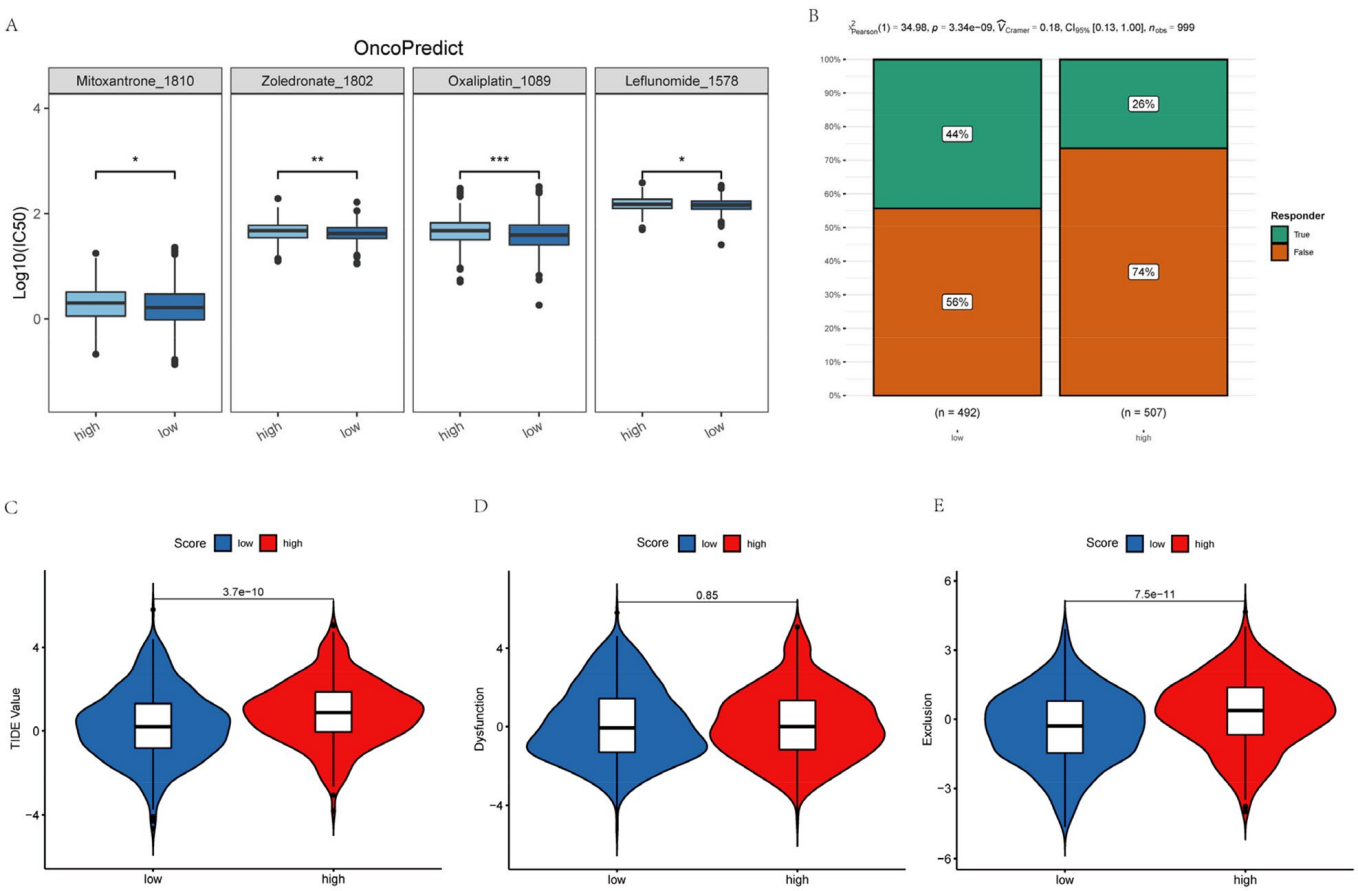
Inflammatory-related gene signature for predicting sensitive chemotherapy drugs and the effect of immunotherapy. (A) OncoPredict package was used to predict sensitive chemotherapy drugs. (B) The likelihood of the clinical response to anti-PD1 therapy for high and low riskscore patients from the TCGA cohorts. True represents immunotherapy responders, while false represents immunotherapy nonresponders. (C–E) The violin plots present of TIDE value, immune dysfunction and immune exclusion in high and low riskscore groups.

To further reveal the relationship of inflammatory-related gene risk-score with chemotherapy and immunotherapy responses among NSCLC patients, this work adopted OncoPredict package for predicting sensitive chemotherapy drugs. NSCLC cases showing increased risk-scores had elevated IC50 with mitoxantrone, zoledronate, oxaliplatin and leflunomide (Figure 7(A)). These means higher risk-score NSCLC patients do not sensitive to these chemotherapeutic drugs. Moreover, NSCLC patients with higher risk-score has lower PD-1/PD-L1 therapeutic response (Figure 7(B)). While NSCLC patients with high risk scores have higher TIDE (Tumor Immune Dysfunction and Exclusion) as well as exclusion score (Figure 7(C–E)). Therefore, NSCLC patients with higher inflammatory-related gene risk-score may not sensitive to immunotherapy.

### Targeting KRT6A sensitive to mitoxantrone and oxaliplatin in NSCLC cells

**Figure 8.**
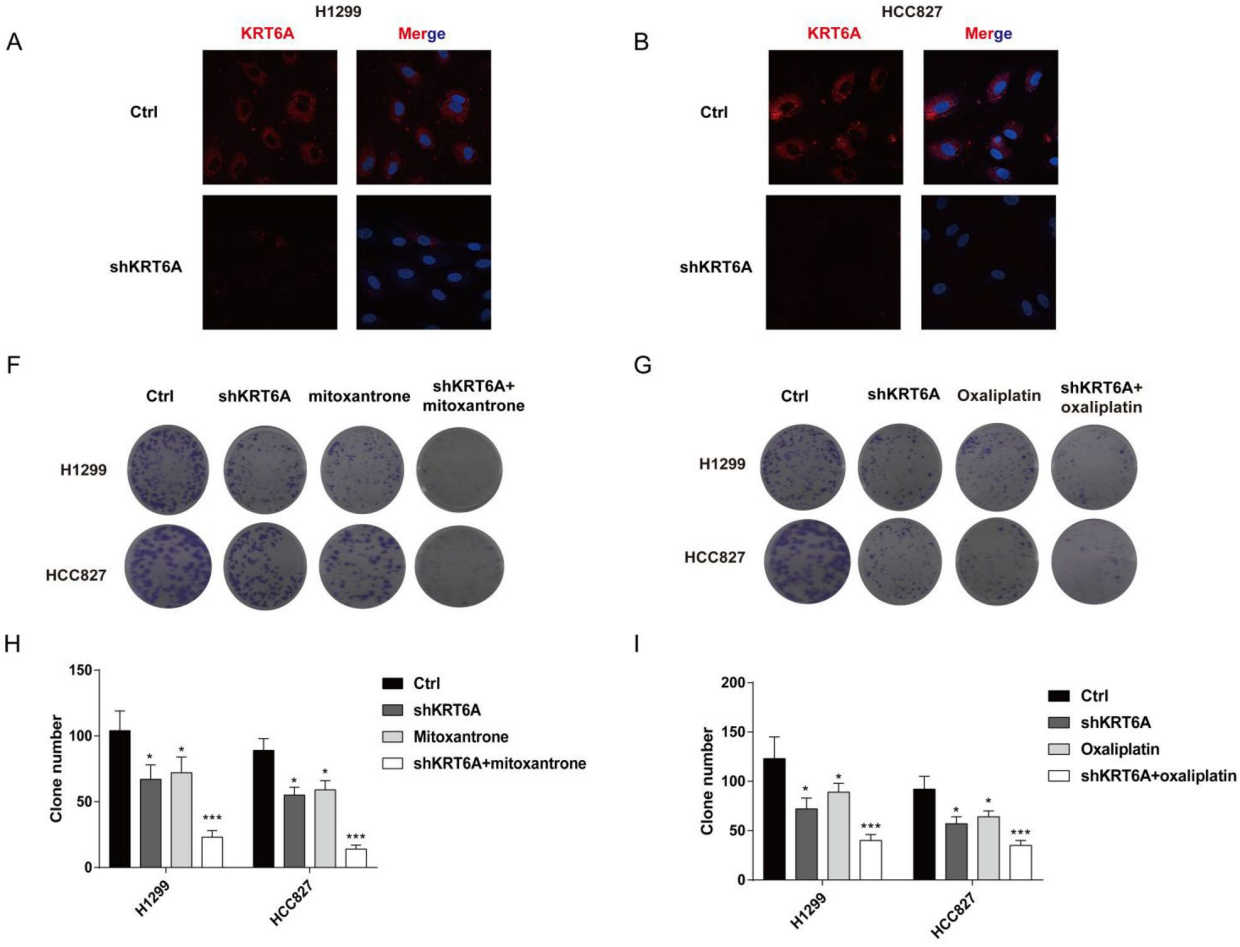
Targeting KRT6A enhance sensitivity of mitoxantrone and oxaliplatin in H1299 and HCC827 cells. (A) Immunofluorescence was used to detect the KRT6A expression after knockdown in H1299 and HCC827 cells. (B,C) Colony forming ability were detected when KRT6A knockdown combined with treatment with mitoxantrone (20 nM) or oxaliplatin (10 μM) in H1299 and HCC827 cells. (D,E) Statistical analysis were performed when KRT6A knockdown combined with treatment with mitoxantrone or oxaliplatin in H1299 and HCC827 cells. (*: vs control group, *p* < .05).

As the risk-score shows as a biomarker for reverse drug resistance in NSCLC, and KRT6A has the highest variable importance for risk-score model. Then, this work detected drug susceptibility after KRT6A knockdown with mitoxantrone or oxaliplatin treatment in NSCLC. Firstly, knockdown KRT6A were detect in H1299 and HCC827 cell by Immunofluorescence (Figure 8(A,B)). At last, the KRT6A knockdown also increased the drug susceptibility when combing the treatment of mitoxantrone or oxaliplatin (Figure 8(C–E)). These result further confirm targeting KRT6A sensitive to mitoxantrone and oxaliplatin in NSCLC.

## Discussion

In this study, we molecular subtypes base on inflammatory-related genes in NSCLC and analyzed the relation with OS in NSCLC cases by publicly available datasets. We identified 8 independent differential genes were selected and included in the random forest prognosis model, included TSLP, TGFBI, SRXN1, RP11 − 680F8.1, KRT6A, CTA-384D8.34, ADORA1 and AC009410.1, and these genes were construct a inflammatory-related gene signature. The NSCLC patients with different inflammatory-related gene signature exhibited diverse functional enrichment together with immune status. At last, this work put forward the prognosis prediction nomogram according to inflammatory-related gene signature, and it was highly sensitive and specific in the prediction of OS of NSCLC. The inflammatory-related genes may be the prognostic and clinical therapeutic response biomarkers for NSCLC. Notably, this is the first molecular subtype prognosis prediction model in NSCLC patients based on inflammatory-related genes.

Persistent infection with carcinogenic irritants such as viruses or microorganisms often activates inflammatory signals, leading to chronic inflammation that promotes tumors and affects the occurrence, progress and metastasis of tumors [15]. Inflammation cells are usually an important source of diverse inflammatory factors, including cytokines like ROS, TNF, IL-1 and IL-6, as well as growth factors that directly or indirectly support the development of tumors. Tumors also help to strengthen this inflammatory cycle [16]. The imbalance of tumor-related genes can cause the overproduction of chemokines and cytokines, and further recruit and activate various immune cells. This work suggested a certain relation among inflammatory-related gene signature within NSCLC and identified inflammatory-related genes for predicting OS and progression of NSCLC, however, its regulation effect remains to be studied.

These above mentioned inflammatory-related genes different express in the stage NSCLC. Thymic lymphopoietin (TSLP), one of the cytokines, shows expression in epithelial cells, and it participates in pathogenic mechanisms of diverse chronic disorders [17]. Previous study found TSLP was a Key factors of maintaining immune homeostasis and regulating inflammatory response of mucosal barrier [17]. Additionally, overexpression of TSLP enhances the interaction between eosinophils and cervical cancer cells to promote angiogenesis in human umbilical vein endothelial cells (HUVECs) [18]. But TSLP’s effect on NSCLC is now clear, our work firstly identify TSLP is down-regulated and is a protective factor in NSCLC. TGFBI is another critical factors for the prognosis of NSCLC. In previous study, methylation of TGFBI promoter predicts dismal prognostic outcome and better chemotherapeutic response among lung cancer cases [19,20]. Keratin 6 A (KRT6A) belongs to the keratin protein family which is a critical component of cytoskeleton in mammalian cells [21]. SRXN1 and KRT6A are the oncogenic genes within NSCLC, which are the candidate markers for smoking exposure and the early diagnostic and prognostic markers for NSCLC among smokers [21]. Moreover, inhibiting ADORA1 expression enhances immune evasion of tumor through modulating ATF3-PD-L1 axis, which is the critical biomarker for ICB response in NSCLC [22]. At last, the eight inflammatory-related genes also predict poor prognosis in NSCLC, conforming to our findings that cases showing low risk scores had extended OS compared with those having high risk scores. Consequently, those above eight inflammatory-related genes may be prognostic markers for NSCLC. Further, we used OncoPredict package for predicting sensitive chemotherapy drugs. NSCLC cases showing high risk scores had increased IC50 with mitoxantrone, zoledronate, oxaliplatin and leflunomide. Thses results further indicate that NSCLC patients with higher inflammatory-related gene risk-score may not sensitive to chemotherapy. At last, knockdown KRT6A in NSCLC cells increased the drug susceptibility when combing the treatment of mitoxantrone or oxaliplatin. These result further confirm targeting KRT6A sensitive to mitoxantrone and oxaliplatin in NSCLC.

To sum up, the present work put forward the new assessment method for inflammatory-related gene signature within NSCLC, and it is essential for predicting NSCLC prognosis. And our study showed that NSCLC patients with different inflammatory-related gene risk-score is the potential biomarker used in chemotherapy and immunotherapy, and targeting KRT6A sensitive to mitoxantrone and oxaliplatin in NSCLC. Mechanisms related to tumor immunity and inflammatory-related genes within NSCLC should be further explored.

## Supplementary Material

Supplemental Material

## Data Availability

Data utilized in the present work can be obtained from corresponding author on request.
