# Supplementary material for: Construction of inflammatory associated risk gene prognostic model of NSCLC and its correlation with chemotherapy sensitivity
Source: Ann Med. 2023 Apr 21;55(1):2200034. doi: 10.1080/07853890.2023.2200034 (PMC10124980; doi:10.1080/07853890.2023.2200034)
Supplement: Supplemental Material [file IANN_A_2200034_SM5328.docx]

| Gene Symbol | Gene Description |
| --- | --- |
| ABCA1 | ATP binding cassette subfamily A member 1 |
| ABI1 | abl interactor 1 |
| ACVR1B | activin A receptor type 1B |
| ACVR2A | activin A receptor type 2A |
| ADM | adrenomedullin |
| ADORA2B | adenosine A2b receptor |
| ADRM1 | adhesion regulating molecule 1 |
| AHR | aryl hydrocarbon receptor |
| APLNR | apelin receptor |
| AQP9 | aquaporin 9 |
| ATP2A2 | ATPase sarcoplasmic/endoplasmic reticulum Ca2+ transporting 2 |
| ATP2B1 | ATPase plasma membrane Ca2+ transporting 1 |
| ATP2C1 | ATPase secretory pathway Ca2+ transporting 1 |
| AXL | AXL receptor tyrosine kinase |
| BDKRB1 | bradykinin receptor B1 |
| BEST1 | bestrophin 1 |
| BST2 | bone marrow stromal cell antigen 2 |
| BTG2 | BTG anti-proliferation factor 2 |
| C3AR1 | complement C3a receptor 1 |
| C5AR1 | complement C5a receptor 1 |
| CALCRL | calcitonin receptor like receptor |
| CCL17 | C-C motif chemokine ligand 17 |
| CCL2 | C-C motif chemokine ligand 2 |
| CCL20 | C-C motif chemokine ligand 20 |
| CCL22 | C-C motif chemokine ligand 22 |
| CCL24 | C-C motif chemokine ligand 24 |
| CCL5 | C-C motif chemokine ligand 5 |
| CCL7 | C-C motif chemokine ligand 7 |
| CCR7 | C-C motif chemokine receptor 7 |
| CCRL2 | C-C motif chemokine receptor like 2 |
| CD14 | CD14 molecule |
| CD40 | CD40 molecule |
| CD48 | CD48 molecule |
| CD55 | CD55 molecule (Cromer blood group) |
| CD69 | CD69 molecule |
| CD70 | CD70 molecule |
| CD82 | CD82 molecule |
| CDKN1A | cyclin dependent kinase inhibitor 1A |
| CHST2 | carbohydrate sulfotransferase 2 |
| CLEC5A | C-type lectin domain containing 5A |
| CMKLR1 | chemerin chemokine-like receptor 1 |
| CSF1 | colony stimulating factor 1 |
| CSF3 | colony stimulating factor 3 |
| CSF3R | colony stimulating factor 3 receptor |
| CX3CL1 | C-X3-C motif chemokine ligand 1 |
| CXCL10 | C-X-C motif chemokine ligand 10 |
| CXCL11 | C-X-C motif chemokine ligand 11 |
| CXCL6 | C-X-C motif chemokine ligand 6 |
| CXCL9 | C-X-C motif chemokine ligand 9 |
| CXCR6 | C-X-C motif chemokine receptor 6 |
| CYBB | cytochrome b-245 beta chain |
| DCBLD2 | discoidin, CUB and LCCL domain containing 2 |
| EBI3 | Epstein-Barr virus induced 3 |
| EDN1 | endothelin 1 |
| EIF2AK2 | eukaryotic translation initiation factor 2 alpha kinase 2 |
| EMP3 | epithelial membrane protein 3 |
| ADGRE1 | adhesion G protein-coupled receptor E1 |
| EREG | epiregulin |
| F3 | coagulation factor III, tissue factor |
| FFAR2 | free fatty acid receptor 2 |
| FPR1 | formyl peptide receptor 1 |
| FZD5 | frizzled class receptor 5 |
| GABBR1 | gamma-aminobutyric acid type B receptor subunit 1 |
| GCH1 | GTP cyclohydrolase 1 |
| GNA15 | G protein subunit alpha 15 |
| GNAI3 | G protein subunit alpha i3 |
| GP1BA | glycoprotein Ib platelet subunit alpha |
| GPC3 | glypican 3 |
| GPR132 | G protein-coupled receptor 132 |
| GPR183 | G protein-coupled receptor 183 |
| HAS2 | hyaluronan synthase 2 |
| HBEGF | heparin binding EGF like growth factor |
| HIF1A | hypoxia inducible factor 1 subunit alpha |
| HPN | hepsin |
| HRH1 | histamine receptor H1 |
| ICAM1 | intercellular adhesion molecule 1 |
| ICAM4 | intercellular adhesion molecule 4 |
| ICOSLG | inducible T cell costimulator ligand |
| IFITM1 | interferon induced transmembrane protein... |
| IFNAR1 | interferon alpha and beta receptor subun... |
| IFNGR2 | interferon gamma receptor 2 |
| IL10 | interleukin 10 |
| IL10RA | interleukin 10 receptor subunit alpha |
| IL12B | interleukin 12B |
| IL15 | interleukin 15 |
| IL15RA | interleukin 15 receptor subunit alpha |
| IL18 | interleukin 18 |
| IL18R1 | interleukin 18 receptor 1 |
| IL18RAP | interleukin 18 receptor accessory protein |
| IL1A | interleukin 1 alpha |
| IL1B | interleukin 1 beta |
| IL1R1 | interleukin 1 receptor type 1 |
| IL2RB | interleukin 2 receptor subunit beta |
| IL4R | interleukin 4 receptor |
| IL6 | interleukin 6 |
| IL7R | interleukin 7 receptor |
| CXCL8 | C-X-C motif chemokine ligand 8 |
| INHBA | inhibin subunit beta A |
| IRAK2 | interleukin 1 receptor associated kinase 2 |
| IRF1 | interferon regulatory factor 1 |
| IRF7 | interferon regulatory factor 7 |
| ITGA5 | integrin subunit alpha 5 |
| ITGB3 | integrin subunit beta 3 |
| ITGB8 | integrin subunit beta 8 |
| KCNA3 | potassium voltage-gated channel subfamily A member 3 |
| KCNJ2 | potassium inwardly rectifying channel subfamily J member 2 |
| KCNMB2 | potassium calcium-activated channel subfamily M regulatory beta subunit 2 |
| KIF1B | kinesin family member 1B |
| KLF6 | Kruppel like factor 6 |
| LAMP3 | lysosomal associated membrane protein 3 |
| LCK | LCK proto-oncogene, Src family tyrosine kinase |
| LCP2 | lymphocyte cytosolic protein 2 |
| LDLR | low density lipoprotein receptor |
| LIF | LIF interleukin 6 family cytokine |
| LPAR1 | lysophosphatidic acid receptor 1 |
| LTA | lymphotoxin alpha |
| LY6E | lymphocyte antigen 6 family member E |
| LYN | LYN proto-oncogene, Src family tyrosine kinase |
| MARCO | macrophage receptor with collagenous structure |
| MEFV | MEFV innate immuity regulator, pyrin |
| MEP1A | meprin A subunit alpha |
| MET | MET proto-oncogene, receptor tyrosine kinase |
| MMP14 | matrix metallopeptidase 14 |
| MSR1 | macrophage scavenger receptor 1 |
| MXD1 | MAX dimerization protein 1 |
| MYC | MYC proto-oncogene, bHLH transcription factor |
| NAMPT | nicotinamide phosphoribosyltransferase |
| NDP | norrin cystine knot growth factor NDP |
| NFKB1 | nuclear factor kappa B subunit 1 |
| NFKBIA | NFKB inhibitor alpha |
| NLRP3 | NLR family pyrin domain containing 3 |
| NMI | N-myc and STAT interactor |
| NMUR1 | neuromedin U receptor 1 |
| NOD2 | nucleotide binding oligomerization domain containing 2 |
| NPFFR2 | neuropeptide FF receptor 2 |
| OLR1 | oxidized low density lipoprotein receptor 1 |
| OPRK1 | opioid receptor kappa 1 |
| OSM | oncostatin M |
| OSMR | oncostatin M receptor |
| P2RX4 | purinergic receptor P2X 4 |
| P2RX7 | purinergic receptor P2X 7 |
| P2RY2 | purinergic receptor P2Y2 |
| PCDH7 | protocadherin 7 |
| PDE4B | phosphodiesterase 4B |
| PDPN | podoplanin |
| PIK3R5 | phosphoinositide-3-kinase regulatory subunit 5 |
| PLAUR | plasminogen activator, urokinase receptor |
| PROK2 | prokineticin 2 |
| PSEN1 | presenilin 1 |
| PTAFR | platelet activating factor receptor |
| PTGER2 | prostaglandin E receptor 2 |
| PTGER4 | prostaglandin E receptor 4 |
| PTGIR | prostaglandin I2 receptor |
| PTPRE | protein tyrosine phosphatase receptor type E |
| PVR | PVR cell adhesion molecule |
| RAF1 | Raf-1 proto-oncogene, serine/threonine kinase |
| RASGRP1 | RAS guanyl releasing protein 1 |
| RELA | RELA proto-oncogene, NF-kB subunit |
| RGS1 | regulator of G protein signaling 1 |
| RGS16 | regulator of G protein signaling 16 |
| RHOG | ras homolog family member G |
| RIPK2 | receptor interacting serine/threonine kinase 2 |
| RNF144B | ring finger protein 144B |
| ROS1 | ROS proto-oncogene 1, receptor tyrosine kinase |
| RTP4 | receptor transporter protein 4 |
| SCARF1 | scavenger receptor class F member 1 |
| SCN1B | sodium voltage-gated channel beta subunit 1 |
| SELE | selectin E |
| SELL | selectin L |
| SELENOS | selenoprotein S |
| SEMA4D | semaphorin 4D |
| SERPINE1 | serpin family E member 1 |
| SGMS2 | sphingomyelin synthase 2 |
| SLAMF1 | signaling lymphocytic activation molecule family member 1 |
| SLC11A2 | solute carrier family 11 member 2 |
| SLC1A2 | solute carrier family 1 member 2 |
| SLC28A2 | solute carrier family 28 member 2 |
| SLC31A1 | solute carrier family 31 member 1 |
| SLC31A2 | solute carrier family 31 member 2 |
| SLC4A4 | solute carrier family 4 member 4 |
| SLC7A1 | solute carrier family 7 member 1 |
| SLC7A2 | solute carrier family 7 member 2 |
| SPHK1 | sphingosine kinase 1 |
| SRI | sorcin |
| STAB1 | stabilin 1 |
| TACR1 | tachykinin receptor 1 |
| TACR3 | tachykinin receptor 3 |
| TAPBP | TAP binding protein |
| TIMP1 | TIMP metallopeptidase inhibitor 1 |
| TLR1 | toll like receptor 1 |
| TLR2 | toll like receptor 2 |
| TLR3 | toll like receptor 3 |
| TNFAIP6 | TNF alpha induced protein 6 |
| TNFRSF1B | TNF receptor superfamily member 1B |
| TNFRSF9 | TNF receptor superfamily member 9 |
| TNFSF10 | TNF superfamily member 10 |
| TNFSF15 | TNF superfamily member 15 |
| TNFSF9 | TNF superfamily member 9 |
| TPBG | trophoblast glycoprotein |
| VIP | vasoactive intestinal peptide |
